# Associations of food consumption, serum vitamins and metabolic syndrome risk with physical activity level in middle-aged adults: the National Health and Nutrition Examination Survey (NHANES) 2005–2006

**DOI:** 10.1017/S1368980015003742

**Published:** 2016-02-17

**Authors:** Jihyun E Choi, Barbara E Ainsworth

**Affiliations:** Exercise Science and Health Promotion, School of Nutrition and Health Promotion, Arizona State University, ASU Mail Code 3020, 500 N. 3rd Street, Phoenix, AZ 85004, USA

**Keywords:** Accelerometer, Metabolic syndrome, Food consumption, Serum vitamin, Physical activity

## Abstract

**Objective:**

To examine the associations of food consumption, serum vitamins and metabolic syndrome risk with physical activity level in middle-aged adults.

**Design:**

Cross-sectional.

**Setting:**

National Health and Nutrition Examination Survey (NHANES) 2005–2006.

**Subjects:**

Adults aged 40–70 years were divided into three groups by tertile of accelerometer-determined steps/d (in men and women, respectively): tertile 1 (sedentary), <6802, <5785; tertile 2 (intermediate), 6802–10698, 5785–9225; tertile 3 (active), ≥10699, ≥9226.

**Results:**

The active men consumed more grain products, fruits and vegetables, whereas the active women consumed more legumes and vegetables, compared with the sedentary group. Serum vitamin concentrations were associated with daily steps in both men and women. Vitamin C, α-carotene, *trans*-β-carotene, *cis*-β-carotene, β-cryptoxanthin, lutein+zeaxanthin, lycopene, γ-tocopherol and vitamin D were significantly associated with daily steps. OR (*P*<0·05) for the sedentary group were 1·52 and 1·61 for low HDL cholesterol, 1·66 and 3·97 for hypertriacylglycerolaemia, 1·02 and 2·73 for abdominal obesity, 1·79 and 1·77 for hyperglycaemia, 1·59 and 1·60 for hypertension, and 1·85 and 2·47 for metabolic syndrome in men and women, respectively.

**Conclusions:**

Those with the highest steps taken showed a more healthful eating profile and a better serum vitamin profile compared with less active adults. Those with the lowest steps taken had greater odds of having metabolic syndrome and its risk components. Probably, daily walking is a marker of a healthful eating profile and increasing daily walking is one of the healthful ways to decrease the metabolic syndrome and its risk components.

The metabolic syndrome (MetS) is a cluster of CHD risk factors: diabetes and elevated fasting blood glucose, abdominal obesity, low HDL cholesterol (HDL-C) and high blood pressure^(^ ^1^ ^–^ ^3^ ^)^. It is estimated that 20–25 % of the world’s adult population and approximately 34 % of the US population have MetS^(^ ^4^ ^)^. Those with elevated MetS risk factors are twice as likely to die from MetS and three times as likely to have a heart attack or stroke compared with people without MetS. In addition, people with MetS have a fivefold greater risk of developing type 2 diabetes^(^ ^5^ ^)^.

According to the American Heart Association^(^ ^6^ ^)^, the following factors have been reported as being directly associated with incident MetS in prospective or retrospective cohort studies: age, low socio-economic status, smoking, low levels of physical activity, intake of soft drinks, Western dietary pattern, heavy alcohol consumption, long-term stress at work, obesity or BMI. Four behavioural risk factors targeted in primary care to prevent and manage non-communicable diseases, including MetS and its sequelae, type 2 diabetes and CVD, are tobacco use, physical inactivity, unhealthy diet and alcohol^(^ ^7^ ^)^. One of the four behavioural risk factors, physical inactivity, is not only a leading risk factor for mortality but also a significant contributor to rising health-care costs. Each year, approximately 3·2 million deaths and 32·1 million disability-adjusted life years, representing about 2·1 % of global disability-adjusted life years, are attributable to insufficient physical activity^(^ ^8^ ^)^. People who are insufficiently physically active have a 20–30 % increased risk of all-cause mortality compared with those who engage in at least 30 min of moderate-intensity physical activity on most days of the week^(^ ^9^ ^)^. Participation in 150 min of moderate physical activity each week or equivalent is estimated to reduce the risk of IHD by approximately 30 %, the risk of diabetes by 27 % and the risk of breast and colon cancer by 21–25 %^(^ ^8^ ^,^ ^9^ ^)^. In the National Health and Nutrition Examination Survey (NHANES) 2007–2008, 32 % of US men and 36 % of US women were obese and an additional 40 % of men and 28 % of women were overweight. About one in twenty Americans had a BMI of >40 kg/m^2^ (class III obesity)^(^ ^10^ ^)^. Compared with adults with a BMI of 20·0–24·9 kg/m^2^, those with a BMI of 30·0–34·9 kg/m^2^ and ≥35·0 kg/m^2^ had 25 % and 44 % higher mean annual total (inpatient and outpatient) health-service costs, respectively^(^ ^11^ ^)^.

Although a large number of studies have reported associations between MetS and physical activity behaviours using questionnaires, little research has been conducted about the relationship of MetS risk with objectively measured physical activity. A literature search using the keywords ‘objectively measured physical activity’, ‘accelerometer’ and ‘MetS’ in PubMed yielded only fourteen articles. Studies reporting associations between daily steps and MetS yielded only two articles^(^ ^12^ ^,^ ^13^ ^)^. The remaining articles reported associations between intensity-related physical activity and MetS. Furthermore, to our knowledge, no studies have been published about the associations of food consumption and serum vitamins with MetS risk stratified by levels of physical activity measured by daily steps taken.

However, diet is indispensible in every day of our lives and one of the four major risk factors for non-communicable diseases^(^ ^7^ ^)^. Also diet can be an immediate factor affecting the action of other factors. This means that diet is a key element in maintaining or increasing physical activity and in disease prevention or treatment. In addition, serum vitamin concentrations are closely connected with physical activity, usual food consumption and health status. For example, low levels of serum vitamins are closely associated with insufficient physical activity, insufficient consumption of fruit and vegetables, and poor health status such as overweight and MetS as well as undernutrition. In this respect, it is valuable to measure these three factors (food consumption, serum vitamin status, MetS risk) by objectively measured physical activity level. Therefore the objective of the present study was to examine the associations of food consumption, serum vitamins and MetS risk with physical activity level in middle-aged adults.

## Methods

### Data collection

The present study used cross-sectional data from the participants of NHANES 2005–2006. NHANES is one of a series of health-related programmes conducted by the Centers for Disease Control and Prevention’s National Center for Health Statistics. The NHANES target population is the civilian, non-institutionalized US population and the survey is a stratified, multistage probability sample design. NHANES 2005–2006, one of the continuous NHANES, refers to the two-year cycles of data produced since 1999. Each cycle is divided into five sections labelled by collection method: Demographics, Dietary, Examination, Laboratory and Questionnaire. All of the NHANES five-section data files can be linked by using the common survey participant identification number. Details of NHANES 2005–2006 can be found online^(^ ^14^ ^)^. We combined the five-section data to analyse associations between level of daily steps and MetS risk and to examine differences in food consumption and levels of serum vitamins by the level of daily steps. Individuals selected for the present study were adults aged 40–70 years and at high risk for MetS. NHANES 2005–2006 contains data for 10348 individuals of all ages. Among the participants, women who were pregnant or lactating and individuals whose accelerometer was not in calibration were excluded. Moreover, missing measurements that existed in Dietary, Examination and Laboratory data were removed. Therefore, the final analytical sample size (of men and women, respectively) for each analysis was as follows: (i) in sociodemographic characteristics and MetS risk analysis, *n* 948 a*n*d *n* 982; (ii) in food group consumption analysis for 2 d dietary data, *n* 838 and *n* 896; and (iii) in serum vitamins analysis, *n* 747–813 and *n* 789–868.

### Accelerometer-determined daily steps

Daily steps (steps/d) were obtained from the physical activity monitor data in the NHANES 2005–2006 Examination section. The physical activity monitor component was added to NHANES in 2003, with NHANES 2005–2006 being the first release of accelerometer-determined step data along with the more commonly collected and reported intensity and duration data based on accelerometer-determined activity counts. The primary objective of the physical activity monitor component is to collect objective information on physical activity. The device used was the ActiGraph AM-7164 (formerly the CSA/MTI AM-7164), manufactured by ActiGraph LLC (Fort Walton Beach, FL, USA). This device is powered by a small watch battery with a small electric signal emitted during movement. The device is programmed to detect and record the magnitude of acceleration or intensity of movement; acceleration data are stored in memory according to a specified time interval. A 1 min time interval or ‘epoch’ was used to aggregate accelerometer data. As the activity monitors are not waterproof, activities such as swimming and water aerobics are not recorded. Therefore, a bias can exist because the physical activity assessment is not for a whole day if the wearer stays in a water environment. However, to account for day-to-day variations and reliably estimate habitual physical activity in adults, Mathews *et al*. suggested 3 to 5 d of monitoring (optimally 7 d)^(^ ^15^ ^,^ ^16^ ^)^. In the present study, participants (948 men and 982 women) who had at least 3 d of accelerometer data were used in the final analysis. Additionally, 98 % of the participants had seven valid days of accelerometer data. A detailed description of the monitors and studies that have used this device is posted on the ActiGraph website^(^ ^17^ ^,^ ^18^ ^)^. Steps were recorded by the ActiGraph accelerometer and summed as total steps taken and steps/min for 24 h (1440 min).

### Metabolic syndrome definition

According to the updated guideline of the US National Cholesterol Education Program Adult Treatment Panel III, MetS was defined as the presence of three or more of the following five components: (i) waist circumference (WC) ≥102 cm for men and ≥88 cm for women; (ii) TAG ≥150 mg/dl or taking medication to treat hypertriacylglycerolaemia; (iii) HDL-C <40 mg/dl for men and <50 mg/dl for women or taking medication to treat low HDL-C; (iv) blood pressure ≥130/85 mmHg or taking medication to treat hypertension; and (v) fasting blood glucose ≥100 mg/dl or taking medication to treat hyperglycaemia^(^ ^19^ ^)^. The MetS data were obtained from the NHANES 2005–2006 Examination, Laboratory and Questionnaire section files.

### Food group consumption

In the dietary recall survey of NHANES 2005–2006, two dietary interviews were administered to all sample persons. The primary dietary interview was administered in person in the mobile examination centre (called ‘the MEC in-person interview’). A follow-up dietary interview was conducted by telephone 3 to 10 d later from the home office (called ‘the phone follow-up interview’). The two non-consecutive days of 24 h dietary recall do not include the same day of the week (if the in-person MEC interview was conducted on Monday, then Monday would not be an available day for scheduling a phone follow-up interview). Using a computer-assisted dietary data entry system, all foods collected from the 24 h dietary recall of the respondents were coded with an 8-digit, US Department of Agriculture food code. Four data files were produced from the information collected in the dietary interview by the 24 h dietary recall method: two Individual Foods files and two Total Nutrient Intake files. Each file includes 1 d of intake data. Therefore, we used the two Individual Foods files to estimate the average daily intake of each food group. We classified all foods reported in the two Individual Foods files (first day: 4052 foods; second day: 3808 foods) into nine food groups according to the US Department of Agriculture food classification: (i) milk and milk products; (ii) meat, poultry, fish and mixtures; (iii) eggs; (iv) legumes, nuts and seeds; (v) grain products; (vi) fruits; (vii) vegetables; (viii) fats, oils and salad dressings; and (ix) sugars, sweets and beverages^(^ ^20^ ^,^ ^21^ ^)^. Then the average consumption of these nine food groups was calculated per person per day for each group by tertile of accelerometer-determined steps/d.

### Serum vitamins

Vitamin C, vitamin B_12_, α-carotene, *trans*-β-carotene, *cis*-β-carotene, β-cryptoxanthin, γ-tocopherol, lutein+zeaxanthin, *trans*-lycopene, total lycopene, vitamin A, vitamin E and vitamin D in the NHANES 2005–2006 Laboratory file were used for the estimation of serum vitamin status by the level of daily steps. Detailed procedures for phlebotomy, subsequent handling of blood samples and analysis of samples, as well as reporting procedures for each of the serum vitamins, can be found online^(^ ^14^ ^)^. The assay methods used for measurement of serum analytes are detailed in the NHANES documentation and included the following: HPLC with electrochemical detection for vitamin C; RIA for serum vitamin B_12_; the two-step Diasorin procedure for vitamin D; and HPLC with photodiode array detection for vitamins A and E, γ-tocopherol and the carotenoids (α-carotene, *trans*-β-carotene, *cis*-β-carotene, β-cryptoxanthin, lutein+zeaxanthin, *trans*-lycopene and total lycopene)^(^ ^14^ ^)^.

### Statistical analysis

Data analysis was performed separately for men and women because it is assumed that men and women have different daily steps^(^ ^22^ ^–^ ^24^ ^)^ and sociodemographic characteristics^(^ ^25^ ^,^ ^26^ ^)^. Three categories of independent variables were created by tertile of accelerometer-determined steps/d (in men and women, respectively): tertile 1 (sedentary), <6802, <5785; tertile 2 (intermediate), 6802–10698, 5785–9225; tertile 3 (active): ≥10699, ≥9226. Means and standard errors were calculated. In sociodemographic characteristics analysis, ANOVA with Bonferroni *post hoc* testing was used for multiple comparisons to determine mean differences. In food group consumption and serum vitamins analysis, univariate general linear model ANCOVA with Bonferroni *post hoc* testing was used. Covariates included age, BMI and total energy intake. Logistic regression was used to estimate the odds ratios and 95 % confidence intervals for MetS risk. Covariates included age, BMI, total energy intake, poverty income ratio, ethnicity and education level, and the active steps/d group was considered to be a reference. All reported *P* values are based on two-sided tests and measured to a significance level of 0·05. The sample weights, WTMEC2YR (full sample 2-year MEC exam weight), WTINT2YR (full sample 2-year interview weight) and WTDR2D (dietary 2-day sample weight), were used in all analyses to obtain unbiased national estimates. Statistical analyses were conducted using the Complex Samples module in the statistical software package IBM SPSS Statistics, version 19.

## Results

### Characteristics of the population

Table 1 shows the sociodemographic characteristics and MetS components by physical activity group and sex. As expected, the daily steps taken were inversely associated with age in men and women. The poverty income ratio was the highest in the intermediate activity group and the lowest in the sedentary group for men and women. For ethnicity, Black men and Black women had the highest prevalence in the sedentary group while White men and Hispanic/Others women had the lowest prevalence in the sedentary group. Conversely, Black men and Black women had the lowest prevalence in the active group. For education, among men, prevalence in the sedentary group decreased with education. The highest prevalence in the active group was observed for men with 9–12 years of education. Among women, the highest prevalence in the sedentary group was observed for those with 9–12 years of education. Prevalence in the active group increased with educational attainment in women.

**Table 1:** Sociodemographic characteristics and MetS components by tertile of daily steps in adults aged 40–70 years, NHANES 2005–2006

	Men								Women					
	Sedentary (<6802 steps/d; *n* 313)		Intermediate (6802–10698 steps/d; *n* 322)		Active (≥10699 steps/d; *n* 313)			Sedentary (<5785 steps/d; *n* 324)		Intermediate (5785–9225 steps/d; *n* 334)		Active (≥9226 steps/d; *n* 324)		
Characteristic	Mean or %	se	Mean or %	se	Mean or %	se	*P* value*	Mean or %	se	Mean or %	se	Mean or %	se	*P* value*
Age (years)	53·2^**a**^	0·0	52·5^**b**^	0·0	51·9^**c**^	0·0	<0·01	54·2^**a**^	0·0	52·2^**b**^	0·0	51·8^**c**^	0·0	<0·01
BMI (kg/m^2^)	31·6^**a**^	0·0	29·1^**b**^	0·0	27·8^**c**^	0·0	<0·01	32·5^**a**^	0·0	30·4^**b**^	0·0	26·5^**c**^	0·0	<0·01
WC (cm)	111·3^**a**^	0·0	103·6^**b**^	0·0	100·3^**c**^	0·0	<0·01	104·9^**a**^	0·0	98·5^**b**^	0·0	89·2^**c**^	0·0	<0·01
HDL-C (mg/dl)	47·2^**a**^	0·0	47·3^**b**^	0·0	53·1^**c**^	0·0	<0·01	57·4^**a**^	0·0	58·8^**b**^	0·0	63·8^**c**^	0·0	<0·01
TAG (mg/dl)	179·5^**a**^	0·0	170·6^**b**^	0·0	146·3^**c**^	0·0	<0·01	185·8^**a**^	0·0	134·4^**b**^	0·0	111·9^**c**^	0·0	<0·01
FBG (mg/dl)	115·4^**a**^	0·0	106·2^**b**^	0·0	104·3^**c**^	0·0	<0·01	115·6^**a**^	0·0	102·5^**b**^	0·0	99·6^**c**^	0·0	<0·01
SBP (mmHg)	128·3^**a**^	0·0	126·9^**b**^	0·0	124·1^**c**^	0·0	<0·01	127·8^**a**^	0·0	124·8^**b**^	0·0	123·0^**c**^	0·0	<0·01
DBP (mmHg)	75·3^**a**^	0·0	75·7^**b**^	0·0	73·6^**c**^	0·0	<0·01	73·5^**a**^	0·0	72·3^**b**^	0·0	73·2^**c**^	0·0	<0·01
PIR	3·2^**a**^	0·0	3·6^**b**^	0·0	3·5^**c**^	0·0	<0·01	2·9^**a**^	0·0	3·6^**b**^	0·0	3·5^**c**^	0·0	<0·01
Ethnicity (%)														
Hispanic/Others	31·1		32·6		36·3		<0·01	22·6		36·3		41·1		<0·01
White	28·3		35·3		36·3			28·3		33·7		38·0		
Black	38·2		33·9		27·8			41·1		35·4		23·5		
Education (%)														
<9 years	36·3		38·1		25·6		<0·01	27·6		37·5		34·9		<0·01
9–12 years	30·7		31·4		37·8			33·4		30·8		35·8		
>12 years	28·4		36·5		35·1			26·5		36·1		37·4		

MetS, metabolic syndrome; NHANES, National Health and Nutrition Examination Survey; WC, waist circumference; HDL-C, HDL cholesterol; FBG, fasting blood glucose; SBP, systolic blood pressure; DBP, diastolic blood pressure; PIR, poverty income ratio. ^a,b,c^Mean values within a row with unlike superscript letters were significantly different (multiple comparisons between tertiles within each sex by Bonferroni *post hoc* test; *P*<0·05). *P value for difference between tertiles within each sex calculated from ANOVA.

BMI, WC and other components of MetS such as HDL-C, TAG, fasting blood glucose and blood pressure were significantly inversely associated with daily steps. Overall, men had higher values than women for WC, and women had higher values than men for BMI, HDL-C and TAG. The increased values for the MetS components seen with lower level of daily steps were greater in women than men. Compared with other components of the MetS, TAG values decreased the greatest with higher daily steps in men and women.

### Food group consumption

As shown in Table 2, compared with the sedentary group, the active men consumed significantly more grain products, fruits and vegetables, whereas the active women consumed significantly more legumes and vegetables.

**Table 2:** Food group consumption by tertile of daily steps in adults aged 40–70 years, NHANES 2005–2006

	Men								Women					
	Sedentary (<6802 steps/d; *n* 262)		Intermediate (6802–10698 steps/d; *n* 286)		Active (≥10699 steps/d; *n* 290)			Sedentary (<5785 steps/d; *n* 286)		Intermediate (5785–9225 steps/d; *n* 307)		Active (≥9226 steps/d; *n* 303)		
Food group (g)	Mean	se	Mean	se	Mean	se	*P* value*	Mean	se	Mean	se	Mean	se	*P* value*
Milk & milk products	270·9	18·4	226·7	16·0	252·0	16·0	NS	214·2	14·5	202·0	13·6	235·8	13·1	NS
Meat, poultry, fish & mixtures	263·6	10·9	250·8	9·5	256·4	9·5	NS	180·4	7·5	179·9	7·0	163·7	6·8	NS
Eggs	28·4	3·3	26·1	2·8	31·0	2·8	NS	21·5	2·3	24·0	2·2	18·8	2·1	NS
Dry beans, peas, other legumes, nuts & seeds	38·9	4·3	35·0	3·8	32·0	3·8	NS	19·5^a^	3·4	23·3^a^	3·2	38·7^b^	3·1	<0·01
Grain products	284·0^a^	12·4	342·6^b^	10·8	345·5^b^	10·8	<0·05	243·0	10·4	265·0	9·7	267·1	9·4	NS
Fruits	151·6^a^	15·1	163·8^a,b^	13·2	202·3^b^	13·1	<0·05	145·9	11·0	153·6	10·3	174·8	10·0	NS
Vegetables	174·9^a^	11·8	236·7^b^	10·3	216·3^b^	10·3	<0·01	173·0^a^	9·2	193·1^a,b^	8·6	207·8^b^	8·3	<0·05
Fats, oils & salad dressings	18·6	1·4	17·2	1·2	17·3	1·2	NS	14·5	1·1	13·7	1·0	15·4	1·0	NS
Sugar, sweets & beverages	2491·1	81·6	2644·5	71·1	2684·6	71·0	NS	2153·7	62·1	2145·5	58·2	2266·5	56·1	NS

NHANES, National Health and Nutrition Examination Survey. Covariates: age, BMI, total energy intake. ^a,b^Mean values within a row with unlike superscript letters were significantly different (multiple comparisons between tertiles within each sex by Bonferroni *post hoc* test; *P*<0·05). *P value for difference between tertiles within each sex calculated from general linear model ANCOVA.

### Serum vitamin status

As shown in Tables 3 and 4, serum vitamin concentrations were significantly associated with daily steps in both men and women. For both sexes, the active group had the highest concentrations and the sedentary group had the lowest concentrations for vitamin C, α-carotene, *trans*-β-carotene, *cis*-β-carotene, β-cryptoxanthin, lutein+zeaxanthin, *trans*-lycopene, total lycopene and vitamin D. γ-Tocopherol was the highest in the sedentary group in men and women. No differences were observed by activity group for vitamin B_12_, vitamin A and vitamin E among men or women.

**Table 3:** Serum vitamin concentrations by tertile of daily steps in men aged 40–70 years, NHANES 2005–2006

	Sedentary (<6802 steps/d)			Intermediate (6802–10698 steps/d)			Active (≥10699 steps/d)			
Serum vitamin	*n*	Mean	se	*n*	Mean	se	*n*	Mean	se	*P* value*
Vitamin C (mg/dl)	242	0·7^a^	0·0	282	0·8^a^	0·0	283	1·0^b^	0·0	<0·01
Vitamin B_12_ (pg/ml)	239	494·8	54·0	277	530·1	46·8	277	539·6	46·8	NS
α-Carotene (µg/dl)	239	3·3^a^	0·4	283	4·2^a,b^	0·4	284	5·3^b^	0·4	<0·01
*trans*-β-Carotene (µg/dl)	239	12·4^a^	1·1	283	16·0^b^	1·0	284	18·0^b^	1·0	<0·01
*cis*-β-Carotene (µg/dl)	223	0·8^a^	0·1	267	1·0^a,b^	0·1	257	1·1^b^	0·1	<0·05
β-Cryptoxanthin (µg/dl)	238	7·6^a^	0·5	281	8·6^a^	0·4	282	10·3^b^	0·5	<0·01
γ-Tocopherol (µg/dl)	239	258·6^a^	8·6	282	232·4^a,b^	7·4	283	216·3^b^	7·3	<0·01
Lutein+zeaxanthin (µg/dl)	239	14·0^a^	0·7	283	16·3^b^	0·6	284	18·9^c^	0·6	<0·01
*trans*-Lycopene (µg/dl)	239	21·5^a^	0·8	283	25·6^b^	0·7	284	26·2^b^	0·7	<0·01
Total lycopene (µg/dl)	239	40·0^a^	1·5	283	47·8^b^	1·3	284	48·9^b^	1·3	<0·01
Vitamin A (µg/dl)	239	66·5	1·2	283	65·4	1·0	284	65·4	1·0	NS
Vitamin E (µg/dl)	239	1269·7	32·9	283	1301·2	28·2	284	1359·5	28·1	NS
Vitamin D (ng/ml)	245	22·4^a^	0·5	283	23·1^a,b^	0·5	285	24·2^b^	0·5	<0·05

NHANES, National Health and Nutrition Examination Survey. Covariates: age, BMI, total energy intake. ^a,b,c^Mean values within a row with unlike superscript letters were significantly different (multiple comparisons between tertiles by Bonferroni *post hoc* test; *P*<0·05). *P value for difference between tertiles calculated from general linear model ANCOVA.

**Table 4:** Serum vitamin concentrations by tertile of daily steps in women aged 40–70 years, NHANES 2005–2006

	Sedentary (<5785 steps/d)			Intermediate (5785–9225 steps/d)			Active (≥9226 steps/d)			
Serum vitamin	*n*	Mean	se	*n*	Mean	se	*n*	Mean	se	*P* value*
Vitamin C (mg/dl)	270	0·8^a^	0·0	292	1·1^b^	0·0	296	1·1^b^	0·0	<0·01
Vitamin B_12_ (pg/ml)	263	527·5	111·0	292	570·8	95·0	293	684·0	94·0	NS
α-Carotene (µg/dl)	272	4·8^a^	0·5	288	5·6^a^	0·4	295	7·6^b^	0·4	<0·01
*trans*-β-Carotene (µg/dl)	272	18·0^a^	1·7	288	23·5^a^	1·5	295	30·5^b^	1·5	<0·01
*cis*-β-Carotene (µg/dl)	251	1·1^a^	0·1	264	1·4^a^	0·1	274	1·9^b^	0·1	<0·01
β-Cryptoxanthin (µg/dl)	271	7·6^a^	0·6	288	10·0^b^	0·5	292	11·8^c^	0·5	<0·01
γ-Tocopherol (µg/dl)	270	262·3^a^	9·5	286	224·2^b^	8·4	295	205·9^b^	8·2	<0·01
Lutein+zeaxanthin (µg/dl)	272	14·8^a^	0·6	288	17·1^b^	0·5	295	18·6^b^	0·5	<0·01
*trans*-Lycopene (µg/dl)	272	21·5^a^	0·7	288	24·6^b^	0·6	295	25·1^b^	0·6	<0·01
Total lycopene (µg/dl)	272	39·8^a^	1·3	288	45·7^b^	1·2	295	46·9^b^	1·2	<0·01
Vitamin A (µg/dl)	272	60·1	1·0	288	60·0	0·9	295	58·4	0·9	NS
Vitamin E (µg/dl)	272	1296·8	34·0	288	1424·4	29·9	295	1410·3	29·5	NS
Vitamin D (ng/ml)	276	20·2^a^	0·6	295	22·8^b^	0·5	297	25·1^c^	0·5	<0·01

NHANES, National Health and Nutrition Examination Survey. Covariates: age, BMI, total energy intake. ^a,b,c^Mean values within a row with unlike superscript letters were significantly different (multiple comparisons between tertiles by Bonferroni *post hoc* test; *P*<0·05). *P value for difference between tertiles calculated from general linear model ANCOVA.

### Metabolic syndrome risk

Table 5 shows odds ratios and 95 % confidence intervals for MetS risk components. The active group was the referent group. The sedentary group had an approximate 1·9- and 2·5-fold risk for MetS in men and women, respectively. Sedentary behaviour was 1·5 to 1·8 times more likely to develop the risks for four of the five components of MetS in men and increased the risks of the five components by 1·6- to 3·9-fold in women. Among participants in the sedentary group, men had the highest risks for hyperglycaemia (OR=1·79) and hypertriacylglycerolaemia (OR=1·66), and women had the highest risks for hypertriacylglycerolaemia (OR=3·97) and abdominal obesity (OR=2·73).

**Table 5:** MetS risk by tertile of daily steps in adults aged 40–70 years, NHANES 2005–2006

	Men						Women					
	Sedentary (<6802 steps/d; *n* 277)		Intermediate (6802–10698 steps/d; *n* 300)		Active (≥10699 steps/d; *n* 299)		Sedentary (<5785 steps/d; *n* 288)		Intermediate (5785–9225 steps/d; *n* 312)		Active (≥9226 steps/d; *n* 303)	
MetS and five components	OR	95% CI	OR	95% CI	OR	95% CI	OR	95% CI	OR	95% CI	OR	95% CI
MetS	1·85	1·84, 1·85	1·29	1·28, 1·29	1·00	Ref·	2·47	2·47, 2·48	1·55	1·54, 1·55	1·00	Ref.
Low HDL-C	1·52	1·51, 1·52	1·45	1·45, 1·45	1·00	Ref.	1·61	1·61, 1·62	1·35	1·35, 1·36	1·00	Ref.
Hypertriacylglycerolaemia	1·66	1·66, 1·67	1·56	1·56, 1·57	1·00	Ref.	3·97	3·96, 3·98	1·68	1·68, 1·69	1·00	Ref.
Abdominal obesity	1·02	1·01, 1·02	0·88	0·88, 0·88	1·00	Ref.	2·73	2·72, 2·74	1·50	1·50, 1·51	1·00	Ref.
Hyperglycaemia	1·79	1·79, 1·79	1·25	1·24, 1·25	1·00	Ref.	1·77	1·77, 1·78	1·50	1·50, 1·51	1·00	Ref.
Hypertension	1·59	1·58, 1·59	1·24	1·24, 1·25	1·00	Ref.	1·60	1·59, 1·60	1·22	1·22, 1·23	1·00	Ref.

MetS, metabolic syndrome; NHANES, National Health and Nutrition Examination Survey; HDL-C, HDL cholesterol; Ref., referent category. Covariates: age, BMI, total energy intake, poverty income ratio, ethnicity, education.

## Discussion

Many studies have been reported that a lack of physical activity is a major risk factor for MetS^(^ ^27^ ^–^ ^29^ ^)^. The present study also found that lower daily steps were significantly associated with higher risk of MetS. Compared with the active group, OR for MetS were elevated for both men (sedentary, OR =1·85; intermediate, OR=1·29) and women (sedentary, OR=2·47; intermediate, OR=1·55). The OR for components of MetS were also significantly higher than 1·0 for sedentary and intermediate activity groups in men and women. This underscores the importance of regular physical activity in the prevention of MetS and its components in men and women. With regard to the risk difference between men and women, previous studies have reported that the association between sedentary behaviours and MetS is stronger in women than in men, and the risk of MetS is increased more in women than in men with prolonged time spent in sedentary behaviours^(^ ^30^ ^–^ ^32^ ^)^. However, it is debatable whether women are more susceptible to developing MetS than men as some studies suggest that men are more likely to develop MetS than women. According to a recent study by Fernádez-Bergés *et al*.^(33)^, in persons aged up to 55 years, MetS is more frequent in men but becomes more frequent in women in persons aged over 65 years.

In addition, the present study showed that there were significant differences between men and women in the OR of the five MetS components. Men had a high risk to develop hyperglycaemia and hypertriacylglycerolaemia, while women had a high risk to develop hypertriacylglycerolaemia and abdominal obesity. In a study of 1958 older adults from the Australian Diabetes Obesity and Lifestyle Study^(34)^, Gardiner *et al.* found that overall sitting time was detrimentally associated with greater risk of having high TAG levels in both sexes, abdominal obesity in women and low HDL-C in men. Also, Travers *et al.* ^(35)^ and Williams *et al.* ^(36)^ reported that the prevalence of diabetes and elevated fasting glucose was higher in men than in women. In the present study, the OR of women for abdominal obesity was two times higher than that of men. Women’s WC itself is naturally smaller than that of men, but the increase was greater in women than in men with lower daily steps (Table 1). According to a WHO report, women have a greater relative risk of CVD at lower WC than do men generally^(37)^. In a follow-up study of 18 892 middle-aged Finnish men and women by Hu *et al.* ^(38)^, the age- and study year-adjusted hazard ratios of CVD across quartiles of WC were 1·0, 1·15, 1·37 and 1·97 in men, and 1·0, 1·41, 1·37 and 2·18 in women, respectively. These results and those observed in the present study indicate that while the mean of WC is smaller in women than men, the variation of WC is greater with lower daily steps and has a greater influence on the development of MetS in women than in men.

In examining the serum vitamin concentrations by daily step levels, higher concentrations of vitamin C, α-carotene, *trans*-β-carotene, *cis*-β-carotene, β-cryptoxanthin, lutein+zeaxanthin, *trans*-lycopene, total lycopene and vitamin D were significantly associated with higher daily steps in both sexes. These vitamins called antioxidants help protect the body against oxidative stress. The oxidative stress induced by free radicals is involved in the aetiology of a wide range of chronic diseases such as CVD, diabetes, renal disease and cancer^(39)^. It has been reported that antioxidant nutrient status and exercise training have an interactive effect on oxidative stress and antioxidant enzyme activities^(^ ^40^ ^,^ ^41^ ^)^. This suggests that eating enough antioxidants in foods or moderate exercise may lead to increased blood vitamins and minerals and decreased oxidative stress, which can prevent development of oxidative stress-related diseases like obesity, MetS, type 2 diabetes and cancer. According to epidemiological studies, serum concentrations of β-carotene and vitamin C were lower in obese individuals in the SUpplémentation en VItamines et Minéraux AntioXydants (SU.VI.MAX) study, which was conducted in 1821 women aged 35–60 years and 1307 men aged 45–60 years^(42)^. Coyne *et al.* ^(43)^ found that mean serum concentrations of α-carotene, β-carotene and sum of five carotenoids (α-carotene, β-carotene, β-cryptoxanthin, lutein and lycopene) were significantly lower in persons with MetS than persons without MetS in a study conducted on 1523 adults aged 25 years and over. α-Carotene, β-carotene and total carotenoids also decreased significantly with increased number of components of MetS. Alipanah *et al.* ^(44)^ determined that low total serum carotenoid (α-carotene, β-carotene, β-cryptoxanthin, lutein+zeaxanthin, lycopene) concentrations were associated with low walking speed and greater decline of walking speed during 3 years of follow-up. Cesari *et al.* ^(45)^ also determined that plasma antioxidant concentrations correlated positively with physical performance and strength in the part of the Invecchiare in Chianti (ageing in the Chianti area; InCHIANTI) study that was conducted in 986 people aged 65 years and older. Also, Shardell *et al*.^(46)^ observed a modest decrease in mortality for the second and third quartiles of lycopene intake in NHANES III. In another NHANES study, Patel *et al*.^(47)^ determined that serum *trans*-lycopene was negatively associated with all-cause mortality. Specifically, higher levels of *trans*-lycopene were associated with a 20 % decreased risk for mortality. Interestingly, in the present study, higher levels of serum vitamin C and serum carotenoids (α-carotene, β-carotene, β-cryptoxanthin, lutein+zeaxanthin, *trans*-lycopene, total lycopene) were significantly associated with higher levels of walking and lower risks of MetS. Those people also consumed more foods rich in vitamin C and carotenoids, such as fruits and vegetables, as shown in Table 2. Namely, the results of our study are closely in agreement with the above findings.

Some studies have been conducted on the relationship among vitamin D, physical activity and multiple pathological conditions including MetS, CVD and type 2 diabetes. Gagnon *et al.* ^(48)^ found that lower serum vitamin D concentration was associated with increased MetS risk and higher WC, serum TAG, fasting blood glucose and insulin resistance. Thomas *et al.* ^(49)^ found that physical activity was positively associated with 25-hydroxyvitamin D (25(OH)D) level and optimal 25(OH)D levels (≥75 nmol/L) substantially lowered all-cause and CVD mortality in individuals with MetS. Also, Ardestani *et al.* ^(50)^ confirmed that there was a significant interaction between 25(OH)D level and self-reported hours of moderate-to-vigorous physical activity and a direct relationship between 25(OH)D levels and VO_2max_ in men and women over a broad age range (20–73 years) and various serum 25(OH)D levels (10–82 ng/ml) regardless of seasonal variations of 25(OH)D. Furthermore, higher levels of physical activity are generally associated with better serum vitamin D status, presumably because more outdoor physical activity can increase sun exposure and vitamin D production in the skin^(16)^. If the above research results are synthesized with the results observed in the present study, physical activity increases serum 25(OH)D level and increased serum 25(OH)D will lead to both increased VO_2max_ and reduced risks for MetS.

Perhaps the most interesting result from the present study is that γ-tocopherol was significantly inversely related with daily steps, unlike other serum vitamins. Significantly lower levels of γ-tocopherol in the active group were observed in both sexes. This result can be explained in two ways: physical activity and physical condition. Chorell *et al.* ^(51)^ detected decreased levels of γ-tocopherol in highly fit individuals, suggesting that this result could be explained by an increased intracellular uptake and utilization of γ-tocopherol in highly fit individuals due to adaption to the oxidative stress during regular physical training. Also, Ford *et al.* ^(52)^ reported that γ-tocopherol concentration was positively associated with concentrations of glucose and glycosylated Hb. In the study of Bates *et al.* ^(53)^ using data from the British National Diet and Nutrition Survey, higher plasma γ-tocopherol was significantly directly correlated with indices of obesity such as body weight, BMI, WC and waist-to-hip ratio. This correlation was higher in adults aged 35 years and over than in young people, especially in women aged 65 years and over. In the analysis of a prospective, nested case–control study conducted by Hak *et al.* ^(54)^, it was shown that men with high plasma γ-tocopherol levels were more likely to have an increased risk of non-fatal and fatal myocardial infarction. Therefore, for the present study, it is plausible that the γ-tocopherol concentration of the active group was the lowest as an adaption to oxidative stress caused by aerobic exercise such as walking and running to increase daily steps. On the other hand, a possible reason why the sedentary group had the highest concentration of γ-tocopherol may be because the sedentary group had some other high-risk condition that can lead to CVD, type 2 diabetes and MetS outcomes. However, other authors have shown that the decreased plasma status of γ-tocopherol and some carotenoids during the acute phase of myocardial infarction normalized the year after the myocardial infarction event in a case–control and follow-up study^(55)^. Their results are contrary to ours. We can interpret this strange result as follows. The mean values of γ-tocopherol in the cases and controls were too low. According to the geometric mean and selected percentile values of serum γ-tocopherol concentration for the total US population aged 6 years and older in NHANES 2005–2006^(56)^, the mean serum γ-tocopherol concentration of control subjects was only about the 5th percentile value^(55)^, whereas the mean serum γ-tocopherol concentrations in the present and aforementioned^(^ ^51^ ^–^ ^54^ ^)^ studies were about the 50th percentile value.

Regarding the food group consumption results, the male active group consumed significantly more grain products, fruits and vegetables and female active group consumed significantly more legumes and vegetables than comparison groups. Accordingly, a recent study of 1359 middle-aged French persons in the SU.VI.MAX study showed that leisure-time physical activity such as walking and gardening was positively associated with eating healthy foods such as fruits and vegetables in both sexes^(57)^. In the Pelotas Birth Cohort Study designed to identify dietary patterns and relationships with sociodemographic and lifestyle factors of 4202 young Brazilian adults, those with a higher adherence to the vegetable/fruit dietary pattern had higher leisure-time physical activity^(^ ^58^ ^)^. Also, a cross-sectional study of older adults aged 70 years and over showed that higher levels of physical activity assessed by accelerometry were significantly correlated with the ease of purchasing fresh fruits, vegetables and low-fat products^(^ ^59^ ^)^. Georgiou *et al*.^(^ ^60^ ^)^ identified that exercisers considered it more important to eat nutritious foods, ate more nutrient-dense low-fat foods, and more frequently met the Food Guide Pyramid-recommended grain and fruit intakes, than non-exercisers. Therefore, it appears that more active people eat more healthy foods and nutritious foods. The importance of fruit and vegetable consumption cannot be overemphasized because fruits and vegetables are an excellent source of various bioactive compounds including vitamins, minerals and antioxidants such as carotenoids, which have a cardioprotective effect. Also, higher levels of dietary fibre in fruits and vegetables may decrease the development of MetS by reducing blood cholesterol and lipid oxidation, and by modulating oxidative stress. All of these things make it clear that the avoidance of sedentary behaviours, healthy eating and good health are mutually connected.

The strength of the present study is that it is unique in identifying differences in food group consumption and serum vitamin status to analyse MetS risks by objectively measured daily steps in a representative sample of the US civilian non-institutionalized population. On the other hand, the weakness of the study is that detailed analysis about food consumption (e.g. separate sweets and beverages from the group of ‘sugar, sweets and beverages’) was not performed. Thus the study was unable to provide information on the detailed food intake affecting serum vitamin levels. A second limitation of the present study is that exact causality of the relationships among food intake, serum vitamin concentrations, daily steps and MetS cannot be suggested because of the cross-sectional design. However, as the primary study objective was to evaluate the association between daily steps and MetS and the risk conditions, and to find some difference in food intake and serum vitamin status by daily steps, the study was effective in providing new information regarding lifestyle behavioural risks for MetS. Most importantly, MetS is lowest and serum vitamin status is highest among those with the highest daily steps. Consequently, it appears that increasing daily walking is a helpful way to decrease MetS and its risk components.
